# Solid Lipid Nanoparticles Administering Antioxidant Grape Seed-Derived Polyphenol Compounds: A Potential Application in Aquaculture †

**DOI:** 10.3390/molecules27020344

**Published:** 2022-01-06

**Authors:** Adriana Trapani, María Ángeles Esteban, Francesca Curci, Daniela Erminia Manno, Antonio Serra, Giuseppe Fracchiolla, Cristóbal Espinosa-Ruiz, Stefano Castellani, Massimo Conese

**Affiliations:** 1 Department of Pharmacy-Drug Sciences, University of Bari “Aldo Moro”, Via Orabona 4, 70125 Bari, Italy; adriana.trapani@uniba.it (A.T.); francesca.curci@uniba.it (F.C.); giuseppe.fracchiolla@uniba.it (G.F.); 2 Department of Cell Biology and Histology, Faculty of Biology, Regional Campus of International Excellence “Campus Mare Nostrum”, University of Murcia, 30100 Murcia, Spain; cespinosa31416@gmail.com; 3 Dipartimento di Matematica e Fisica “E. De Giorgi”, University of Salento, 73100 Lecce, Italy; daniela.manno@unisalento.it (D.E.M.); antonio.serra@unisalento.it (A.S.); 4 Department of Biomedical Sciences and Human Oncology, University of Bari “Aldo Moro”, 70124 Bari, Italy; stefano.castellani@uniba.it; 5 Department of Medical and Surgical Sciences, University of Foggia, 71122 Foggia, Italy; massimo.conese@unifg.it

**Keywords:** solid lipid nanoparticles, grape seed–derived extract, drug delivery, X-ray diffraction, antioxidant activity, fish cells

## Abstract

The supply of nutrients, such as antioxidant agents, to fish cells still represents a challenge in aquaculture. In this context, we investigated solid lipid nanoparticles (SLN) composed of a combination of Gelucire^®^ 50/13 and Precirol^®^ ATO5 to administer a grape seed extract (GSE) mixture containing several antioxidant compounds. The combination of the two lipids for the SLN formation resulted in colloids exhibiting mean particle sizes in the range 139–283 nm and zeta potential values in the range +25.6–43.4 mV. Raman spectra and X-ray diffraction evidenced structural differences between the free GSE and GSE-loaded SLN, leading to the conclusion that GSE alters the structure of the lipid nanocarriers. From a biological viewpoint, cell lines from gilthead seabream and European sea bass were exposed to different concentrations of GSE-SLN for 24 h. In general, at appropriate concentrations, GSE-SLN increased the viability of the fish cells. Furthermore, regarding the gene expression in those cells, the expression of antioxidant genes was upregulated, whereas the expression of *hsp70* and other genes related to the cytoskeleton was downregulated. Hence, an SLN formulation containing Gelucire^®^ 50/13/Precirol^®^ ATO5 and GSE may represent a compelling platform for improving the viability and antioxidant properties of fish cells.

## 1. Introduction

**Figure 1:**
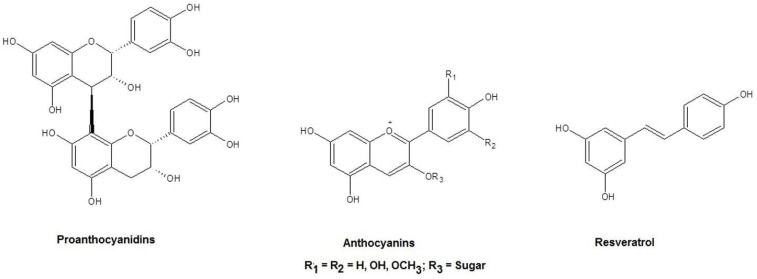
Chemical structures of the main compounds found in grape seed extract (GSE).

To improve fish nutrition, to date, different diet protocols have been proposed, and in each of them, the presence of antioxidant substances has been crucial to ensure optimal fundamental metabolic processes. Apart from single antioxidant molecules such as vitamin E and glutathione, which are already included in fish diet protocols, natural mixtures that include antioxidant compounds can also be of interest for fish nutrition if they are adopted as an entire extract without any separation of the active principles. In this context, we previously studied a mixture based on Apulian grape seed extract (GSE) for its favorable antioxidant and anti-inflammatory effects in human cells due to the relevant pro-anthocyanidin and polyphenol content (Figure 1; in [1,2]). With regard to pro-anthocyanidins contained in GSE, previous studies in the area of fish immunology elucidated that, once excessive fat accumulation takes place in fish, with consequent hepatic lipid metabolism disorder, then GSE allows hypolipidemic and potential anti-inflammatory effects in the liver, as occurred, for instance, in grass carp [3]. Moreover, the antioxidant effects of GSE were recently evaluated in a zebrafish embryo model [4]. However, to the best of our knowledge, no attention has been paid to investigating which type of pharmaceutical dosage form can enhance the administration of GSE using fish cell lines.

In this context, different approaches for the delivery of antioxidant agents were reviewed, evidencing the relevance and the feasibility of the production of colloidal carriers, such as nanoparticles, liposomes, and nanosized lipid particles, for this purpose [5,6]. Among the colloidal carriers, solid lipid nanoparticles (SLN) have been shown to have several benefits, including enhanced safety and stability, and controlled drug release, and can be prepared on a large scale according to scale-up industrial guidelines. In our laboratory, we have already investigated SLNs made of a single lipid, namely Gelucire^®^ 50/13, for uptake in fish cell lines in order to ascertain the possible immunological applications, and by ex vivo investigations, the biocompatibility of such a synthetic lipid was assessed [7,8,9]. From a chemical viewpoint, it could be assumed that using two or more lipids may be better than using only one. In this sense, Precirol^®^ ATO5 is a suitable candidate to be assayed with another lipid. In the literature, Precirol^®^ ATO5 has been combined with other lipids, such as the liquid Transcutol^®^ for paediatric drug administration [10], and it was blended with Compritol^®^ 888 ATO to achieve an SLN for lycopene vectorization [11]. Precirol^®^ ATO5 is based on mono-, di-, and triglycerides of palmitostearic acids (C16 or C18), and the diester fraction accounts for 40–60% of the total mass; whereas Gelucire^®^ 50/13 is composed of mono-, di-, and triglycerides with polyethylene glycol(PEG) residues. According to the manufacturer’s instructions, both in veterinary and human medicine, Precirol^®^ ATO5 and Gelucire^®^ 50/13 are approved, and, indeed, from an excipient regulatory status, Precirol^®^ ATO5 is recognized as GRAS degree and it belongs to the Japanese Standard of Food Additives; whereas Gelucire^®^ 50/13 is approved as a US Food Additive [12,13].

To gain insight into the vector comprising Precirol^®^ ATO5 and Gelucire^®^ 50/13, several studies of the solid state were carried out, namely FT-IR spectroscopy, differential scanning calorimetry (DSC), X-ray diffraction, and Raman spectroscopy.

On the basis of this knowledge, the aim of this study was to assess if GSE administered via modified drug delivery systems, such as nanoparticles rather than conventional dosage forms, could achieve an immunomodulation role in two fish cell lines obtained from gilthead seabream and European sea bass. In the present work, we have attempted to formulate SLN using a lipid mixture, rather than one single lipid, i.e., forming the lipid matrix structure in the presence of Precirol^®^ ATO5 and Gelucire^®^ 50/13. Finally, concerning the biological evaluation of GSE-SLN, the cell viability and the expression of different selected gene codes for the structural proteins involved in cell movement were determined in SAF-1 and DBL-1 cell lines. The results arising from these investigations are reported and discussed below.

## 2. Results

### 2.1. Physico-Chemical Properties of SLN

Table 1 shows the main physico-chemical properties of GSE-SLN. In detail, the particle size of all types of GSE-SLN was found in the range 139–283 nm, with a slightly broader size distribution, as shown by the PDI range values (0.44–0.59). All types of GSE-SLN were smaller than unloaded SLN, whose mean diameter was 486 ± 5 nm. The largest particles herein studied are GSE-SLN_(6mg)_-adsorbing GSE, but their zeta potential value is similar to the that of GSE-SLN_(6mg)_, perhaps because the adsorption layer of GSE, instead of shielding the negative charges of the lipid matrix, induces some structural modifications in the SLN, leading to the exposure on the surface of a higher number of such charges. As for the zeta potential of these nanocarriers, they resulted in the range −25.6–−43.4 mV indicative of a good colloidal stability.

**Figure 2:**
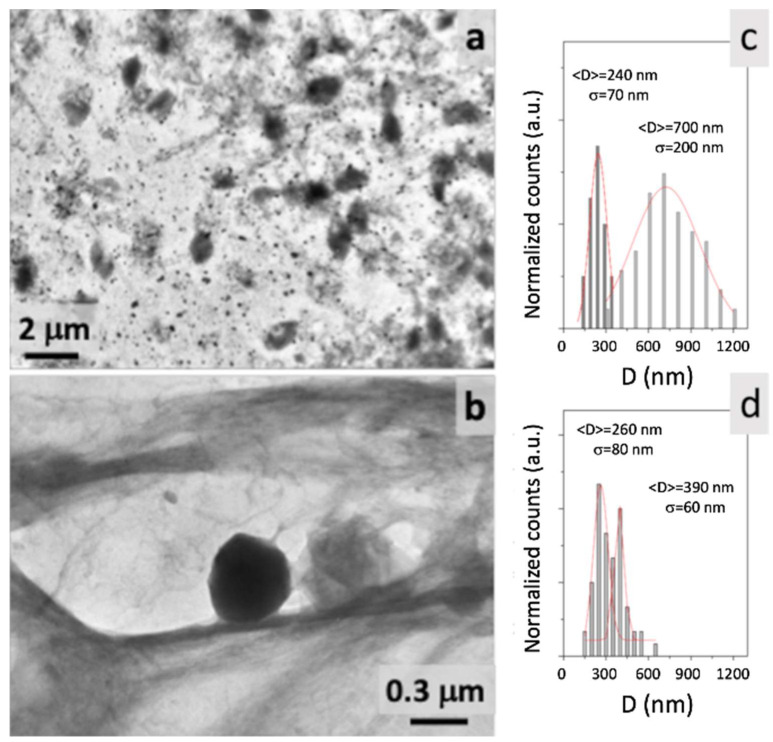
Transmission electron spectroscopy (TEM) images of: GSE-SLN_(6mg)_ (**a**); GSE-SLN_(6mg)_-adsorbing GSE (**b**); and related particle diameter distribution (**c**,**d**). Feret diameters were measured for each sample.

Furthermore, from the data reported in Table 1, high percentages of GSE entrapment were obtained (i.e., 49.7–74.6%) via the use of a lipid matrix where Gelucire^®^ 50/13 and Precirol^®^ ATO5 were combined and, notably, the GSE-SLN_(6mg)_-adsorbing GSE formulation was found to be the one loading the highest amount of the GSE mixture in comparison with our previous work [2]. As shown in Figure 2, to visualize SLN administering GSE, transmission electron spectroscopy (TEM) morphology was examined (Figure 2), and in the case of GSE-SLN_(6mg)_, an oval shape (Figure 2a) was detected rather than the spherical shape of GSE-SLN_(6mg)_-adsorbing GSE (Figure 2b). Moreover, GSE-SLN_(6mg)_ and GSE-SLN_(6mg)_-adsorbing GSE showed a bimodal particle distribution (Figure 2c,d). Importantly, GSE-SLN_(6mg)_ showed a very pronounced bimodal distribution with a clear separation between smaller diameter SLN (<Standard deviation> = 240 nm, S.D. = 70 nm) and larger diameter SLN (<Standard deviation> = 700 nm, s = 200 nm). GSE-SLN_(6mg)_-adsorbing GSE showed a less evident bimodal distribution; in this case, the difference between smaller diameter SLN (<Standard deviation> = 260 nm, S.D. = 80 nm) and larger diameter SLN (<Standard deviation> = 390 nm, S.D. = 80 nm) were much less pronounced, perhaps suggesting a stabilizing action due to the adsorbed GSE.

### 2.2. Solid-State Studies

**Figure 3:**
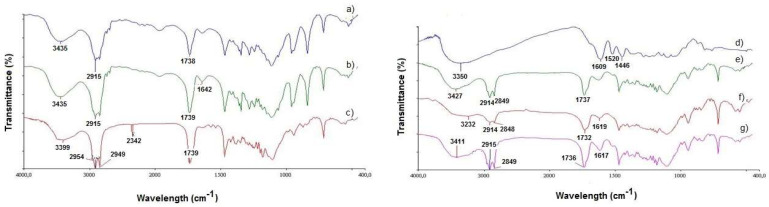
**Left panel**: FT-IR spectra of pure Gelucire^®^ 50/13 (**a**); Gelucire^®^ 50/13/Precirol^®^ ATO5 blend (**b**); and unloaded SLN (**c**). **Right panel**: FT-IR spectra of pure GSE (**d**); GSE-SLN_(6mg)_ (**e**); GSE-SLN_(12mg)_ (**f**); and GSE-SLN_(6mg)_-adsorbing GSE (**g**).

Multiple studies of the solid-state of GSE-SLN were carried out to gain a deeper insight into their organization. With regard to FT-IR spectra (Figure 3), the peaks at 3435 cm^−1^ and 1738–1739 cm^−1^ (Figure 3a–c), and 1732–1737 cm^−1^ (Figure 3b,d) could be assigned to partially hydrated Gelucire^®^ 50/13 [14,15], evidencing the external localization of such lipids in the SLN structure.

Indeed, when the FT-IR spectra of GSE-SLN were examined (as seen in Figure 3a), the characteristic band attributable to GSE at 1609 cm^−1^ was shifted at 1619 cm^−1^ and 1617 cm^−1^ for GSE-SLN_(12mg)_ and GSE-SLN-adsorbing GSE, respectively (Figure 3c,d).

**Figure 4:**
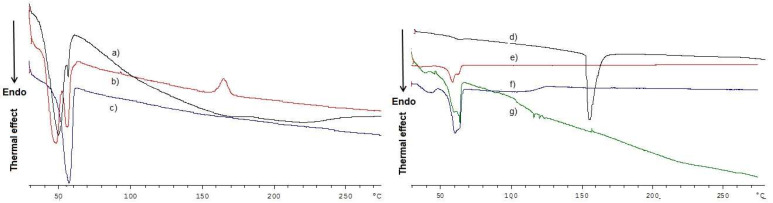
**Left panel**: DSC thermograms of pure Gelucire^®^ 50/13 (**a**); Gelucire^®^ 50/13/Precirol^®^ ATO5 blend (**b**); and unloaded SLN (**c**). **Right panel**: DSC thermograms of pure GSE (**d**); GSE-SLN_(6mg)_ (**e**); GSE-SLN_(6mg)_-adsorbing GSE (**f**); and GSE-SLN_(12mg)_ (**g**).

Regarding DSC thermograms (Figure 4a), the GSE-SLN_(6mg)_-adsorbing GSE thermogram is the only one where small endothermic peaks at 114 °C and 118 °C are shown, and as pure GSE melting point is at 160 °C, they can be attributed to the shift of the endothermal peak of the extract because the esothermal peak at 160 °C resembles one of the Gelucire^®^ 50/13/Precirol^®^ ATO5 blends. Furthermore, as previously seen for the SLN containing GSE based on pure Gelucire^®^ 50/13 [2], in the DSC thermograms of GSE-SLN_(6mg)_ and GSE-SLN_(12mg)_, no peak ascribable to GSE was detected (Figure 4b,d), suggesting that the molecular encapsulation of the extract occurred in these formulations. In the range 47–56 °C, either in the unloaded SLN or in GSE-containing SLN, endothermal signals were recorded, and they can be attributed to the Gelucire^®^ 50/13/Precirol^®^ ATO5 mixture. This assessment is based on the DSC peaks recorded when the Gelucire^®^ 50/13/Precirol^®^ ATO5 blend underwent a calorimetric run (Figure 4b), and it is also corroborated by the fact that the drop point of Precirol^®^ ATO5 is well known to be in the range 53–57 °C, according to the manufacturer instructions.

### 2.3. X-ray Diffraction and Raman Spectroscopy

**Figure 5:**
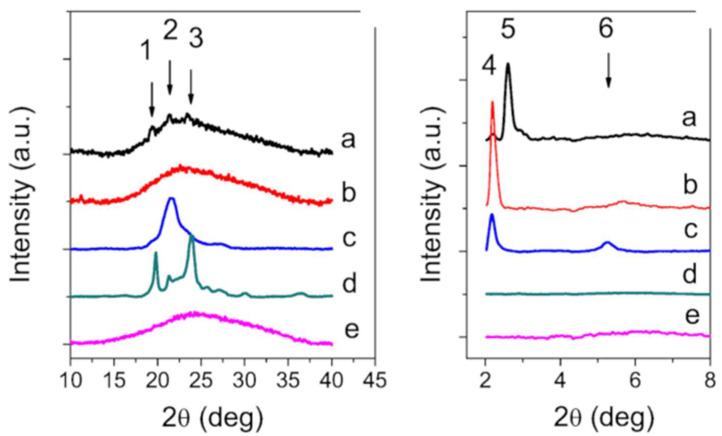
X-ray diffraction spectra of: GSE-SLN_(6mg)_ (**a**); GSE-SLN_(6mg)_-adsorbing GSE (**b**); Precirol^®^ ATO5 (**c**); Gelucire^®^ 50/13 (**d**); and GSE (**e**), in the wide angle range 10–40 deg (**left panel**) and in the small angle range 2–5 deg (**right panel**).

To gain insight into the structural characterization of SLN, the X-ray diffraction patterns of Precirol^®^ ATO5, Gelucire^®^ 50/13, GSE, GSE-SLN_(6mg)_, and GSE-SLN_(6mg)_-adsorbing GSE are shown in Figure 5. The diffraction patterns of Gelucire^®^ 50/13 revealed diffraction maxima at 2q = 19.2, 21.2, and 23.4 deg. These peaks correspond to the lattice spacing 0.46, 0.42, and 0.38 nm, respectively, and are indicative of the crystalline nature of the substance. The X-ray diffractograms of Precirol^®^ ATO5 show large peaks in the small-angle range at 2q = 2.16, 5.25 deg (5 and 6 in Figure 5(right panel)), and 21.2 deg (2 in Figure 5). These reflections correspond to the lattice spacings 4.18, 1.69, and 0.42 nm, respectively. According to the literature [16], the main polymorphic forms of triacylglycerols are α, β′, and β. The α-form is a hexagonal sub-cell with a short spacing of 0.42 nm, the β′-form [17] is an orthorhombic perpendicular sub-cell with short spacings of 0.42–0.43 and 0.37–0.40 nm, and the β-form is a triclinic parallel sub-cell with a short spacing of 0.46 nm [18]. The peaks observed at small angles (0° < θ < 5°) allow us to measure the thickness of the lamellar structures, which corresponds to the longitudinal stacking, and it is possible to deduce whether the stackings correspond to 2 L or 3 L organizations (Figure 5).

The peak at 2q = 2.5 deg corresponds to the stacking 3L_002_ of α phase [19] and leads to a distance of approximately d = 3.5 nm; the peak at 2q = 2.2 deg corresponds to the stacking 2L_002_ of β’ phase and leads to a distance of about d = 4.0 nm [20]. Moving from these measurements, GSE-SLN_(6mg)_ shows a faint peak at 19.2, 21.2, and 23.4 deg superimposed to a large band due to very short-range crystallinity material, together with a faint peak at 2q = 2.2 deg and strong peaks at 2q = 2.5 deg (stacking α + β’ polymorphic form), and GSE-SLN_(6mg)_-adsorbing GSE, with the exception of a peak at 2q = 2.2 deg (stacking 3L_002_ of β’ phase), completely loses any ordered contribution.

**Figure 6:**
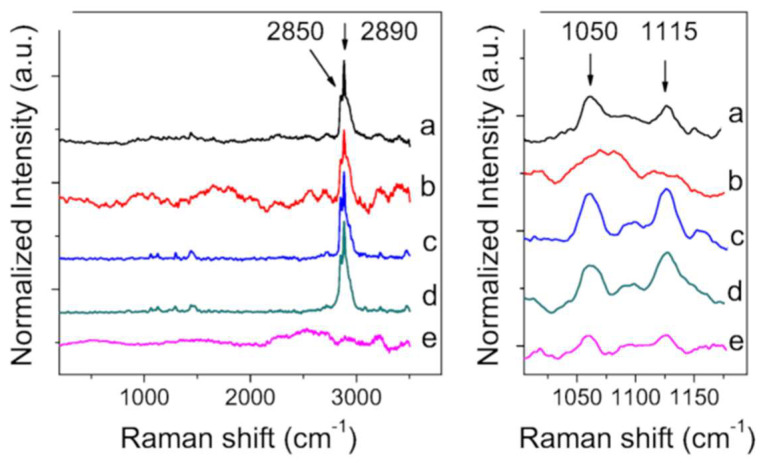
Survey of Raman spectra acquired from: GSE-SLN_(6mg)_ (**a**); GSE-SLN_(6mg)_-adsorbing GSE (**b**); Precirol^®^ ATO5 (**c**); Gelucire^®^ 50/13 (**d**); and GSE (**e**), from different systems (**left panel**) and an enlargement relative to the 1000–1200 cm^−1^ spectral region (**right panel**).

Figure 6 and Table 2 display a survey of Raman spectra for the analysed bulk materials and SLN. The spectral region relative to the 1000–1200 cm^−1^ is primarily related to C-C stretching motions [21]. Both frequency differences and relative intensity changes for these vibrational modes can be used to monitor specific conformational changes in the hydrocarbon chains. The 1100 cm^−1^ region, in particular, has been shown to be a superposition of the C-C stretching modes for segments of all-trans hydrocarbon conformations. An increase in the intensity of the 1115 cm^−1^ band relative to the intensities of the 1050 cm^−1^ transitions is indicative of a greater fluidity within the hydrocarbon chains, so the increase in the 1115 cm^−1^ band originates from the increased intramolecular disorder in the systems.

The region around 3000 cm^−1^ of the Raman spectrum consists of a large number of overlapping peaks, containing both fundamental CH-stretch vibrations and Fermi resonance bands.

The CH_3_ symmetric stretching modes appear in the 2870–2880 cm^−1^ spectral region, with a Fermi resonance (FR) component in the 2930–2940 cm^−1^ region. The peaks in the 2950–2970 cm^−1^ spectral region are the CH_3_ out-of-plane and in-plane methyl antisymmetric stretches [22].

The methylene vibrations at approximately 2850, 2880, 2900, and 2930 cm^−1^ are sensitive to conformational changes as well as intermolecular interactions of the alkyl chains of lipids. The ν_a_(CH_2_) antisymmetric stretch is coupled to rigid rotations–torsional vibrations so that it broadens considerably with temperature, and increases continuously in frequency from 2880 cm^−1^ to 2900 cm^−1^ as gauche conformers are introduced. The ν_s_(CH_2_) symmetric stretch contains three components, centred at 2852 cm^−1^, 2900 cm^−1^, and 2928 cm^−1^, due to extensive Fermi resonance interactions with overtones of the bending modes and is affected by intra- and intermolecular interactions [23].

The relative intensities of these peaks change notably with changes in hydration state, packing, and conformational order. To utilize this spectral sensitivity toward the lipid environment, several spectral parameters have been used in the literature that empirically describe the order of the lipid layers. The peak height ratio I_2890_/I_2850_ has been used as a marker for chain packing and conformational disorder, where higher values indicate a higher ordering of the chains [24].

### 2.4. Antioxidant Activity of GSE-SLN

The antioxidant activity of GSE-SLN was determined and the data are summarized in Table 2. GSE-SLN_(12mg)_ showed higher values of antioxidant activity than GSE-SLN_(6mg)_ or GSE-SLN_(6mg)_-adsorbing GSE. Furthermore, the total antioxidant values for GSE-SLN_(6mg)_-adsorbing GSE were slightly lower than those observed for GSE-SLN_(6mg)_ (Table 3).

### 2.5. Viability Assay

**Figure 7:**
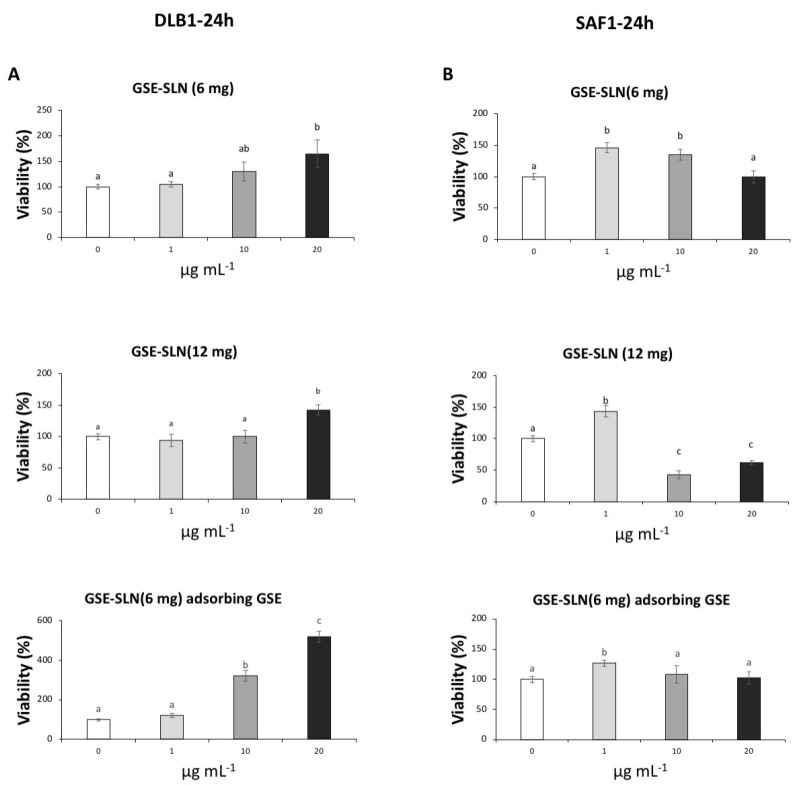
Cytotoxicity of DLB-1 (**A**) and SAF-1 cells (**B**) exposed to different concentrations of GSE-SLN (0, 1, 10, and 20 µg mL^−1^) for 24 h. Bars represent the mean ± SEM (*n* = 6). Statistically significant differences (ANOVA; *p* < 0.05) were denoted using different letters.

The effects of GSE-SLN_(6mg)_, GSE-SLN_(12mg)_, and GSE-SLN_(6mg)_-adsorbing GSE on the viability of SAF-1 and DLB-1 cells were evaluated by 3-(4,5-dimethylthiazol-2-yl)-2,5-diphenyltetrazolium bromide(MTT) (Figure 7). Results from the cytotoxicity test showed that incubation of DLB-1 cells with the highest concentration (20 μg mL^−1^) of GSE-SLN_(6mg)_, GSE-SLN_(12mg)_, and GSE-SLN_(6mg)_-adsorbing GSE significantly increased the cell viability (164.6 ± 27.2%; 142.5 ± 8.4%; and 520.5 ± 27.9%, respectively, *p* < 0.05). These results are in agreement with previous reports describing that cell viability could increase after exposure to plant extracts rich in antioxidant compounds. For example, SAF-1 cells showed increased viability after oregano aqueous extracts, which are demonstrated to have a rich antioxidant profile [25,26,27]. Moreover, incubation with 10 μg mL^−1^ of GSE-SLN_(6mg)_-adsorbing GSE significantly increased the cell viability (321.6 ± 27.3%, *p* < 0.05), meaning that, with a lower concentration of GSE (namely GSE-SLN_(6mg)_), cell viability is reached to a higher degree than GSE-SLN_(12mg)_. The viability results seem to indicate that the SLN antioxidants present in GSE-SLN_(6mg)_-adsorbing GSE particles arrived at cells in higher concentrations than when the cells were incubated with GSE-SLN_(12mg)._

On the other hand, the lowest concentration (1 μg mL^−1^) of GSE-SLN_(6mg)_, GSE-SLN_(12mg)_, and GSE-SLN_(6mg)_-adsorbing GSE significantly increased the SAF-1 viability (146.0 ± 7.9%; 143.6 ± 14.7%; and 126.8 ± 5.2%, respectively, *p* < 0.05). These results seem to indicate that SAF-1 cells are more sensitive to these kinds of molecules than DLB-1 cells. In fact, higher concentrations of GSE, such as 10 and 20 μg mL^−1^ of GSE-SLN_(12mg)_ significantly decreased the viability of SAF-1 cells (42.6 ± 6.1% and 61.5 ± 3.2%, respectively, *p* < 0.05). These results are also consistent with previous studies that demonstrated that exposure to extracts with a high antioxidant profile could increase or decrease the viability of SAF-1 cells depending on their concentrations and nature [25,26].

### 2.6. Gene Expression

**Figure 8:**
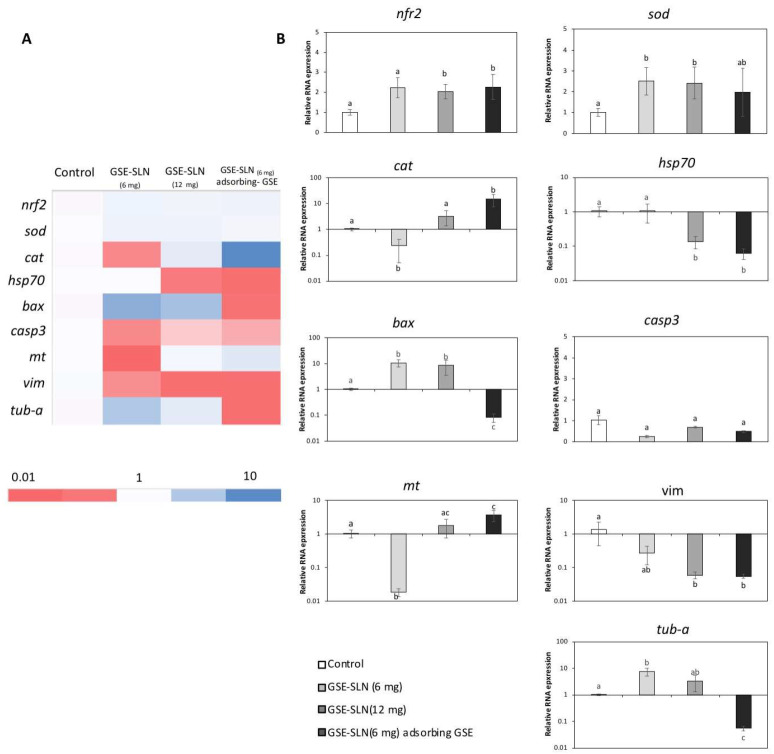
(**A**) Heat-map of the nine differentially expressed genes on DLB-1 cells after GSE-SLN incubation for 24 h. Dark blue: upregulation; red: downregulation. (**B**) Relative gene expression of nine genes (*Nrf2*, *sod*, *cat*, *Hsp70*, *bax*, *casp3*, *mt*, *vim*, and *tubulin α* (*tub-a*)) from DLB-1 cells exposed to 0 (control) or 20 µg mL^−1^ of loaded particles (SLN and SLN-adsorbing GSE) for 24 h. Bars represent the mean ± SEM (*n* = 5). Statistically significant differences (ANOVA; *p* < 0.05) were denoted using different letters.

**Figure 9:**
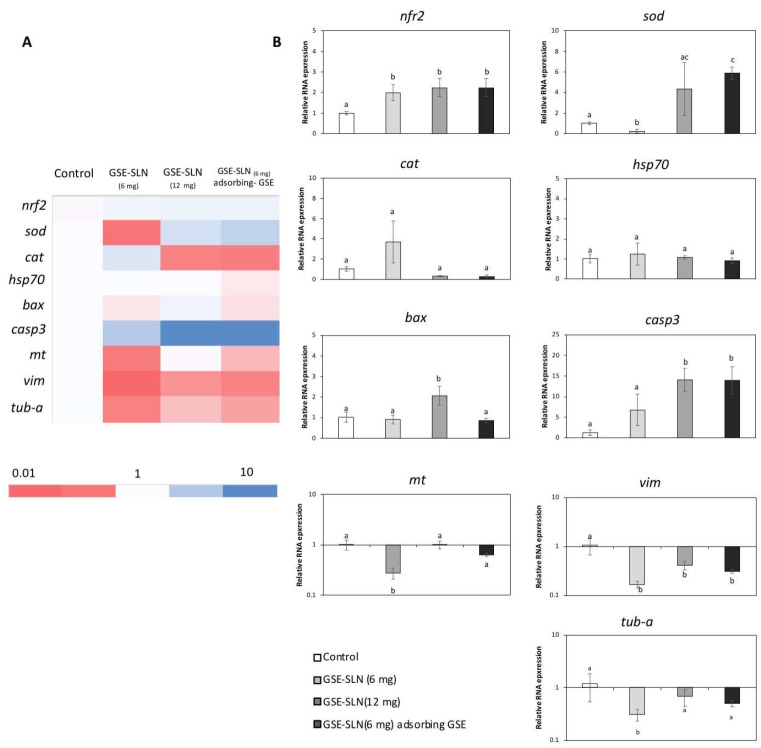
(**A**) Heat-map of the nine differentially expressed genes on SAF-1 cells after GSE-SLN incubation for 24 h. Dark blue: upregulation; red: downregulation. (**B**) Relative gene expression of nine genes (*Nrf2*, *sod*, *cat*, *Hsp70*, *bax*, *casp3*, *mt*, *vim*, *tubulin α* (*tub-a*)) from SAF-1 cells exposed to 0 (control) or 20 µg mL^−1^ of loaded particles (SLN and SLN-adsorbing GSE) for 24 h. Bars represent the mean ± SEM (*n* = 5). Statistically significant differences (ANOVA; *p* < 0.05) were denoted using different letters.

The expression of genes related to antioxidant defence (*Nrf2*, *cat*, and *sod*), stress (*Hsp70*), apoptosis (*bax* and *casp3*), detoxification and antioxidant defence (*mt*), and the cytoskeleton (*vim* and *tub-a*) was evaluated on SAF-1 and DLB1 cells after GSE-SLN_(6mg)_, GSE-SLN_(12mg)_, and GSE-SLN_(6mg)_-adsorbing GSE incubation for 24 h (Figure 8 and Figure 9, Table 4 and Table 5).

## 3. Discussion

Searching for orally approved delivery systems such as solid dispersions [28] and cyclodextrins [29], capable to supply micro and macronutrients for fish growth is still a challenge for fish immunology researchers as. The reason is that, once administered *in vivo*, some of them can fail, leading to the loss of their cargo prior to target immunocompetent fish cells [30,31]. Among antioxidant agents, the whole GSE mixture has been studied for its beneficial effects on fish cells [3,4], Moreover, even the isolated polyphenols arising from GSE; namely, resveratrol was found to inhibit both lipopolysaccharide-induced and endogenous eicosanoid production [32], and polyphenol-enriched extract was seen to decrease the oxidative stress and extend the life span of medaka fish [33].

The safe and effective use of nanoparticles in biology and medicine requires in-depth knowledge, down to the molecular level, of how nanoparticles interact with cells in a physiological environment [34]. Until now, the relevance of GSE nanoparticles for administration to gilthead seabream and European sea bass cell lines has never been investigated. These two model cell lines were herein selected as they were obtained from two of the most important marine fish species farmed in the Mediterranean area. In fact, cell cultures are considered as a feasible approach to implementing the “3R principle”: replacement, reduction, and refinement of animal usage. For the purpose of designing nanoparticles for GSE supply to fish cells, three different types of SLN containing GSE were prepared to employ the Gelucire^®^ 50/13/Precirol^®^ ATO5 blend. From a technological viewpoint, Precirol^®^ ATO5 is well recommended as an excipient for taste masking, offering excellent anti-friction properties, and is ideal for capsule filling. On the other hand, Gelucire^®^ 50/13 acts as a water-dispersible surfactant, forming fine (micro)emulsions and, as is the case with Precirol^®^ ATO5, high biocompatibility is ensured both in the human and veterinary fields. Two of the three formulations of SLN herein presented were conceived with different starting amounts of GSE (i.e., GSE-SLN_(6mg__)_ and GSE-SLN_(12mg)_). Furthermore, the loading of GSE at two different doses (i.e., 6 and 12 mg of GSE) was thought to increase the vectorization of the mixture due to the SLN carrier, while, at the same time, increasing the GSE loading from 6 to 12 initial milligrams in the cargo seemed to be a way to reduce the frequency of administration of the particulate SLN delivery system to the fish cells. The relatively large dimensions of the control SLN could depend on the adoption of two lipids forming the matrix (i.e., Gelucire^®^ 50/13 and Precirol^®^ ATO5). On the other hand, when SLN were loaded with GSE, a reduced mean diameter of these nanocarriers was noted, particularly for those charged with 12 mg of GSE where a size of 139 ± 15 nm was observed. It may be due to the encapsulated GSE, which induces some structural modifications in the SLN structure such as a conformational modification of the PEG moieties occurring in Gelucire^®^ 50/13, leading to SLN shrinkage and exposure of negative charges on the surface of SLN. An intermediate mean diameter between unloaded SLN and GSE-loaded SLN was observed for GSE-SLN_(6mg)_ adsorbing GSE (i.e., 283 ± 32 nm) which causes an increase of mean diameter from 208 ± 21 nm to 283 ± 32 nm. It may be ascribed to the adsorption layer of GSE surrounding SLN. To understand the zeta potential values of observed for GSE-SLN, it is also possible to invoke the lipid composition based on the combination of Gelucire^®^ 50/13 with Precirol^®^ ATO5 taking into account that SLN containing GSE, and only Gelucire^®^ 50/13 as lipid matrix exhibited an external surface charge equal to −14.5 ± 2.0 mV [1]. As previously pointed out, the biggest particles are GSE-SLN adsorbing GSE, but their zeta potential value is similar to the one of GSE-SLN_(6mg)_. It may suggest that in the case of GSE-SLN_(6mg)_ the negative charges on the surface of SLN are more densely located on the surface while, in the case of GSE-SLN_(12mg)_, they are looser and the negative charge density on the surface of these particles is lower. Finally, from TEM observations, indirect information on adsorption was also provided as the nanoparticles were seen to cling to form cluster-shaped complexes.

To understand how the solid-state organization can influence the behavior of the nano-system SLN once incubated with gilthead seabream and European sea bass, first FT-IR spectra and DSC thermograms were acquired. The external localization of Gelucire^®^ 50/13 as derived by FT-IR spectra in our study is in good agreement with the interpretation by Jeon et al. [35], who proposed that the molecular assembly between Precirol^®^ ATO5 and Gelucire 50/13^®^ when the Gelucire^®^ 50/13 to Precirol^®^ ATO5 mass ratio is lower than in our case leads to the fact that Gelucire^®^ 50/13 polar molecules might be located over the surfaces of Precirol^®^ ATO5 molecules, and the lipophilic moieties of Gelucire^®^ 50/13 intercalate between the molecules of Precirol^®^ ATO5. From the FT-IR spectrum of GSE-SLN_(6mg)_-adsorbing GSE, it could be deduced that the adsorption process as described in Section 4.2 also determines, to some extent, the external localization of GSE and, indeed, the corresponding DSC thermogram could reinforce such a hypothesis. Hence, the molecular encapsulation of GSE in the SLN arising from the DSC thermograms of SLN (where no endothermic peak ascribable to GSE is detected) also helps us to understand the high percentages of association efficiency (AE) found for these carriers. Notably, for the GSE-SLN herein described, the molecular distribution of GSE in the lipid matrix derived from DSC thermograms is also consistent with X-ray analysis and is also in agreement with the concept that the amorphous state could contribute to the increased carrying capacity of the active ingredient in the SLN [36]. In parallel, the decrease in the crystalline state of the lipids detected by X-ray diffraction in the SLN has often been observed and indicates the incorporation of the active principles in the SLN [37]. Finally, when Raman spectra were acquired, the intensity ratios of the Raman peaks analysed showed a strengthening of the intermolecular bonds in the layer and between the layers, which is also associated with a more pronounced decrease in long-range crystallinity in GSE-SLN_(6mg)_-adsorbing GSE than in GSE-SLN_(6mg)_.

Furthermore, the exposure of GSE-SLN to DLB-1 and SAF-1 cells allowed us to clarify the genetic processes involved in cell viability and cell defence. First, the expression of antioxidant genes such as *Nrf2*, *cat*, and *sod* was significantly affected by the SLN exposure. NRF2 is a transcription factor that is activated in response to a wide range of oxidative and electrophilic stimulations, including radical oxygen species (ROS) and some chemical agents [38], and it promotes the expression of the antioxidant gene response (such as catalase or superoxide dismutase, which codify the antioxidant enzymes involved in the detoxification of free radicals) and phase II enzymes [39]. In our experiment, *Nrf2* expression was upregulated with respect to the control on DLB-1 and SAF-1 cells after GSE-SLN exposure. These results indicated that GSE-SLN exposure activated the antioxidant response elements that prepared cells against pro-oxidative future events. Increments of *Nrf2* expression after antioxidant exposure both in vivo [40,41] and in vitro [42] have been described. Interestingly, *cat* and *sod* expression in the cells trend to be upregulated with respect to the control after GSE-SLN exposure, although the response depends on the cell type used in the assays (SAF-1 or DLB-1 cells), as was also observed in the viability results.

The management of *Hsp70* expression is an optimal example of a cellular defence mechanism developed to protect the organisms against diverse categories of damages (e.g., high temperature, toxins, and ROS) [43]. Since *HSP70* is considered as a stress marker [44], the decrease in *Hsp70* expression in DLB-1 cells seems to indicate an improvement in the cell welfare parameters, as it has been suggested in vitro [45]. However, no significant changes in *Hsp70* expression were observed on the SAF-1 cells after being incubated with the particles, with respect to the values observed for control samples.

We also monitored the Bax protein trend, where the Bax protein is a member of the Bcl-2 family that promotes apoptosis [46]. The incubation with GSE-SLN significantly affected *bax* expression on the DLB-1 and SAF-1 cells. In fact, the DLB-1 cells showed significant upregulation induced by GSE-SLN_(6mg)_ and GSE-SLN_(12mg)_ exposure (*p* < 0.05), while *bax* expression was significantly downregulated after GSE-SLN_(6mg)_-adsorbing GSE incubation for 24 h (*p* < 0.05). On the other hand, the SAF-1 cells showed the *bax* expression to be downregulated compared with the control group after GSE-SLN_(12mg)_ exposure (*p* < 0.05). Interestingly, the *bax* expression increases on the DLB-1 and SAF-1 cells were not perfectly correlated with the cell viability, except in the case of the SAF-1 cells exposed to GSE-SLN_(12mg)._ Concomitantly, caspase-3 is an executioner protease that stimulates the death receptor extrinsic and mitochondrial intrinsic apoptosis pathways [47]. The *casp3* expression was increased on the SAF-1 cells exposed to GSE-SLN_(12mg)_ and GSE-SLN_(6mg)_-adsorbing GSE, which could be correlated with the decrease in viability observed on the SAF-1 cells after GSE-SLN_(12mg)_ exposure. No significant changes were observed in the *casp3* expression on the DLB-1 cells.

In addition to other antioxidants systems (such as catalase, superoxide dismutase, glutathione, and Zinc ions), metallothionein plays a key role against heavy metal toxicity [48], although it has been proposed to have other functions, such as the sequestration of ROS, radical nitrogen species (RNS), or electrophiles [49]. Both the DLB-1 and SAF-1 cells exposed to GSE-SLN_(6mg)_ showed a decreased *mt* expression, compared with the control cells (*p* < 0.05). However, contrarily, the DLB-1 cells exposed to GSE-SLN_(6mg)_-adsorbing GSE showed a significantly up-regulated *mt* expression (*p* < 0.05). These results are also consistent with the hypothesis that the GSE-SLN_(6mg)_-adsorbing GSE increase antioxidant defence, as described above. Overall, the amount of GSE entrapped in the SLN, once slowly released, elicits a biological effect as was observed for the other SLNs with the same or a higher amount of GSE. These observations, again, point to the acquisition of the same or higher biological effects with lower amounts of GSE, including improvements in skin wound healing, keeping in mind that the SAF-1 cells were obtained from fins [50].

Finally, we also focused on cytoskeleton function, as it is well known that it plays an important role in many cellular processes, including apoptosis, considering that both vimentin and tubulin have been located in apoptotic body formation [51,52]. In general, herein, both vimentin and tubulin gene expression was downregulated on the DLB-1 and SAF-1 cells after exposure to GSE-SLN_(6mg)_, GSE-SLN_(12mg)_, and GSE-SLN_(6mg)_-adsorbing GSE. Overall, although only an in vitro study was performed in the present work, future in vivo studies are planned to investigate the pharmacokinetic properties of the bioactive compounds.

## 4. Materials and Methods

### 4.1. Materials

The grape seed extract was kindly provided by Farmalabor (Canosa di Puglia, Italy). It was obtained after the acetone/water extraction of artificially dried seeds of *Vitis vinifera* L., leading to the final content of pro-anthocyanidins ≥95.0%. According to the manufacturer’s instructions, the grape seed extract also contained 13–19% as total percentage of catechin and epicatechin. Tween^®^85 and the salts used for buffer preparation were purchased from Sigma-Aldrich (Milan, Italy). Gelucire^®^ 50/13 and Precirol^®^ ATO5 were gifted by Gattefossè (Milan, Italy). Throughout this work, double-distilled water was used. All other chemicals were of reagent grade and the different companies are detailed together with the methodology.

### 4.2. Preparation of SLN

The SLNs were prepared from Precirol^®^ ATO5 and Gelucire^®^ 50/13, adopting the melt homogenization procedure, following Jeon et al.’s method with slight modifications [53]. The association of GSE to the SLNs containing Gelucire^®^ 50/13/Precirol^®^ ATO5 followed different procedures: (i) Gelucire^®^ 50/13 (20 mg) and Precirol^®^ ATO5 (50 mg) were co-melted at 80 °C (higher than the melting points of all the lipids) and, in a separate vial, the surfactant (Tween^®^85, 22 mg) and 1.37 mL of double-distilled water were also heated at 80 °C. In the aqueous phase, 6 mg (or 12 mg) of GSE was dispersed under homogenization at 12,300 rpm for 2 min with an UltraTurrax model T25 apparatus (Janke and Kunkel, Germany) and let to equilibrate for 30 min at 80 °C. Afterwards, the aqueous phase was mixed with melted Gelucire^®^/Precirol^®^ ATO5, and the emulsion was homogenized at 12,300 rpm for 2 min by UltraTurrax prior to carrying out centrifugation at 16,000× *g*, for 45 min (Eppendorf 5415D, Germany). The SLN prepared to start from 6 mg and 12 mg of GSE were herein indicated as GSE-SLN_(6mg)_ and GSE-SLN_(12mg)_, respectively; and (ii) a suspension of GSE-SLN_(6mg)_ (0.5 mL), obtained as reported above in (i), was incubated with 1 mL of GSE aqueous solution (concentration of 1 mg/mL) at room temperature for 3 h under light protection and mild stirring (50 oscillations/min). When the incubation time was over, the mixture was then centrifuged at 16,000× *g*, for 45 min and the supernatant was discarded. The SLNs prepared to start from 6 mg of GSE were herein indicated as GSE-SLN_(6mg)_-adsorbing GSE. All pellets resulting from centrifugation were used for the following studies. Unloaded SLNs were prepared following the same procedure described above in (i) without any addition of any GSE to the aqueous phase [8,9,54].

### 4.3. Physic-Chemical Characterization of SLN

Particle size and polydispersion index (PDI) for the SLNs were evaluated using a Zetasizer Nano ZS (ZEN 3600, Malvern, UK) apparatus following photon correlation spectroscopy (PCS) mode. Particle size and PDI were measured after dilution 1:1 (*v*/*v*) with double-distilled water, while the zeta-potential value was determined after dilution of sample 1:20 (*v*/*v*) with KCl (1 mM, pH 7) described in [55]. GSE quantification was assessed by HPLC analysis as previously reported in [2] using an HPLC apparatus consisting of a Waters Model 600 pump (Waters Corp., Milford, MA, USA), a Waters 2996 photodiode array detector, and a 20 μL loop injection autosampler (Waters 717 plus). A Synergy Hydro-RP (25 cm × 4.6 mm, 4 μm particles; Phenomenex, Torrance, CA, USA) column in conjunction with a precolumn C18 insert as a stationary phase was used for the analyses, and the elution of the column in isocratic mode took place at a flow rate of 0.7 mL/min. The mobile phase was composed adopting 0.02 M potassium phosphate buffer, pH 2.8: CH_3_OH 70:30 (*v*/*v*), providing a GSE retention time equal to 12 min. For the GSE, calibration curve linearity (R^2^ > 0.999) was checked over the range of concentrations equal to 100–50 mg/mL, and for concentrations lower than 50 mg/mL, a fluorometer apparatus (Varian Cary Eclipse, Mulgrave, Australia, excitation wavelength: 560 nm; emission wavelength: 583 nm; slits: 2.5 nm) was used. When a fluorometric assay was employed, the linearity was checked over the range of concentrations equal to 50–2.5 mg/mL.

To determine the GSE association efficiency (AE) in the SLN, freeze-dried particles were cleaved upon enzymatic digestion operated by carboxyl ester hydrolase as previously described [2,37]. The enzyme was dissolved at 12 I.U./mL in phosphate buffer (pH 5) and aliquots of lyophilized SLN in the range 1–2 mg were incubated with 1 mL of the enzyme solution for 30 min in an agitated (40 rpm/min) water bath set at 37 °C (Julabo, Milan, Italy). The samples were then centrifuged (16,000× *g*, 45 min, Eppendorf 5415D) and the resulting supernatant was injected in HPLC for GSE content evaluation. The AE% of GSE in the SLN was calculated as follows:

AE% = GSE in the supernatant after enzymatic assay with esterase/Total GSE × 100

where the total GSE is intended as the initial amount of GSE used for the SLN preparation. Each measurement was performed in triplicate.

### 4.4. Transmission Electron Spectroscopy (TEM)

For the GSE containing SLN, TEM observations were also carried out. The morphology and dimensions of the SLNs were determined by cryogenic transmission electron microscopy (Cryo-TEM). All observations were performed using a Hitachi 7700 electron microscope, at a temperature of 105 K and an acceleration voltage of 100 kV. The procedure for vitrifying the samples (as previously described in [56]) can be summarized as follows. A drop of suspension containing the nanocarriers was deposited on copper grids covered with an amorphous carbon film. After removing the excess solution with buffer paper, the sample was vitrified by immersion in liquid ethane maintained just above its freezing point. The sample was then transferred to the Gatan 626 cryo holder. The sample was protected against atmospheric conditions during the entire procedure to prevent the formation of ice crystals. The digital images were acquired with an AMT-XR-81 camera and processed with the EMIP software. Counting and size distribution of the nanoparticles were obtained by processing the obtained TEM images. Twenty fields, randomly chosen, were taken into consideration for each sample, and the morphology and particle size of the particles present in randomly selected areas on the basis of the count of 500 particles for each sample was determined.

### 4.5. Solid State Studies

FT-IR spectroscopy: FT-IR spectra were obtained using powders of 2–5 mg of lyophilized SLN (Lio Pascal 5P, Milan, Italy). Such powders were milled with KBr discs prior to acquire spectra from a Perkin Elmer 1600 FT-IR spectrometer (Perkin Elmer, Milan, Italy). The analysis was carried out at room temperature (r.t.) in the range of 4000–400 cm^−1^ at a resolution of 1 cm^−1^ [57].

DSC: for bulk lipids, unloaded SLN, and GSE-SLN, DSC runs were performed on a Mettler Toledo DSC 822e. The instrument was calibrated with indium for melting point and heat of fusion, and in each run, the heating rate of 5 °C/min was used in the range of 25–275 °C. About 5 mg of lyophilized samples were taken in the standard aluminum sample pans for analysis, and an empty pan was used as reference in each case. The analyses were performed under nitrogen purge; triple runs were carried out on each sample [28,58].

X-ray diffraction: X-ray diffraction spectra were acquired with a MiniFlex Rigaku diffractometer, operating in step-scan mode and equipped with a Cu Kα source (wavelength λ = 0.154 nm) at 30 kV and 100 mA. The X-ray diffraction data were collected in the Bragg-Brentano geometry, from 2 to 8 deg and from 10 to 40 deg, at a scanning speed of 0.02 deg/s.

Raman Spectroscopy: Raman scattering measurements were obtained by a Renishaw spectrometer equipped with a Leica metallographic microscope. The instrumentation included a 514.5 nm air-cooled Argon ions laser source and 1800 lines/mm lattice/monochromator with a RenCam CCD detector that assured a resolution of 1 cm^−1^. The analysis of the obtained data was performed using Renishaw Wire 2.0 software.

### 4.6. Total Antioxidant Activity

The total antioxidant activity (TAA) was evaluated in the SLNs using a method based on the ability of the antioxidants in the sample to reduce the radical cation of 2,20-azino-bis-3-(ethylbenzothiazoline-6-sulphonic acid) (ABTS), determined by the decoloration of ABTS^+^, and measuring the quenching of the absorbance at the wavelength of 730 nm (BMG Labtech, Fluostar Omega, UK) [59]. This activity was calculated by comparing the values of the sample with a standard curve of ascorbic acid, and expressed as ascorbic acid equivalents (mmol) per milligram of SLN.

### 4.7. Cell Lines Culture

Two cell lines, SAF-1 from gilthead seabream and DLB-1 from European sea bass, were used throughout the study. The established SAF-1 cell line [60] was seeded in 75-cm^2^ plastic tissue culture flasks (Nunc, Denmark) and cultured at 25 °C in an atmosphere with 85% relative humidity using a L-15 Leibowitz medium supplemented with 10% foetal bovine serum (FBS, Sigma–Aldrich), 2 mM L-glutamine, 100 µg/mL streptomycin, and 100 U/mL penicillin. The subculture was carried out according to standard trypsinization methods (0.25% trypsin/0.53 mM EDTA, Sigma–Aldrich). The cells were centrifuged (200× *g*, 10 min) and the viability determined using the trypan blue exclusion test. The SAF-1cells were plated in 48-well plates at 2.5 × 10^5^ cell/well and cultured overnight at 25 °C with 85% relative humidity and 5% CO_2_ in the incubator chamber.

#### 4.7.1. Cell Monolayers DLB-1

The DLB-1 cells were grown at 25 °C in a L-15 Leibovitz medium containing 0.16% NaCl, 15% foetal bovine serum (FBS), 20 mM HEPES, 2 mM glutamine, penicillin (100 IU/mL), and streptomycin (100 μg/mL) and subcultured by trypsinization every week. The cells were centrifuged (200× *g*, 10 min) and the viability determined using the trypan blue exclusion test [61,62]. The cells were plated in 48-well plates at 2.5 × 10^5^ cell/well and cultured overnight at 25 °C with 85% relative humidity and 5% CO_2_ in the incubator chamber.

#### 4.7.2. In Vitro Incubation of Fish Cell Lines with Particles

To determine whether GSE-SLN affected the viability of the SAF-1 and DLB-1 cells, the cells were incubated without (control) or with different concentrations of loaded particles (GSE-SLN_(6mg)_, GSE-SLN_(12mg)_, and GSE-SLN_(6mg)_-adsorbing GSE) (1, 10, and 20 µg/mL of culture medium, respectively) at 25 °C with 85% relative humidity for 24 h. All the trials were performed using six replicates.

### 4.8. Viability Assay

The cell viability was evaluated using the MTT (3-(4,5-dimethylthiazol-2-yl)-2,5-diphenyltetrazolium bromide; Sigma-Aldrich) colorimetric assay based on the reduction of the yellow soluble tetrazolium salt into a blue, insoluble formazan product by the mitochondrial succinate dehydrogenase [1]. For this, the SAF-1 and DLB-1 cells were washed and incubated with 200 μL/well of culture medium containing 1 mg/mL of MTT. After 4 h of incubation at 25 °C, the wells were washed, the formazan solubilized, and the absorbance at 570 nm and 690 nm was determined in a microplate reader (BMG Labtech, Fluostar Omega, UK). Blanks consisted of wells without cells.

### 4.9. Gene Expression

The SAF-1 and DLB1 cells were plated on uncoated 12-well culture dishes at a density of 5 × 10^5^ cells/well and incubated until 100% confluence. The cells were exposed in culture medium to 0 (control) or 20 µg mL^−1^ of loaded particles (SLN and SLN-adsorbing GSE) using 3 wells for each one and incubated for 24 h. The relative gene expression on the cells was then evaluated using real-time PCR and the 2^−^^ΔΔ^^CT^ method [63]. 

Total RNA was extracted from the cells using TRIzol reagent (Invitrogen; Berlin, Germany) following the manufacturer’s instructions. The RNA was then treated with DNase I (Invitrogen) to remove genomic DNA contamination. Complementary DNA (cDNA) was synthesized from 1 mg of total RNA using the SuperScriptIV reverse transcriptase (Invitrogen; Berlin, Germany) with an oligo-dT18 primer. The expression of 14 selected genes was analysed by real-time PCR, performed with an ABI PRISM 7500 instrument (Applied Biosystems; Waltham, CA, USA) using SYBRGreen PCR Core Reagents (Applied Biosystems; Waltham, CA, USA), as previously described [64]. These genes code for structural proteins involved in antioxidant response, cell stress, apoptosis, and cell movement (nuclear factor erythroid 2-related factor 2 (*nrf2*), superoxide dismutase (*sod*), catalase (*cat*), heat-shock protein 70 (*hsp70*), BCL2 Associated X, apoptosis regulator (*bax*), caspase 3 (*casp3*), metallothionein (*mt*), vimentine (*vim*), and tubulin alpha (*tub-a*)). For each mRNA, the gene expression was corrected by the median of the β-actin and 18-S expression content in each sample. The primers used are shown in Table 4 and Table 5. In all cases, each PCR was performed with triplicate samples.

### 4.10. Statistical Analyses

For physic-chemical properties and size stability studies, statistical analyses were carried out using Prism Version 4, GraphPad Software Inc. (San Diego, CA, USA). Data were expressed as either mean ± SD. Multiple comparisons were based on one-way analysis of variance (ANOVA) with either Bonferroni’s or Tukey’s post hoc test, and differences were considered significant when *p* < 0.05.

For biological studies, statistical differences among the groups were assessed by one-way ANOVA analyses, followed by the Bonferroni or Games –Howell test, depending on the homogeneity of the variables. The normality of the variables was confirmed by the Shapiro–Wilk test and the homogeneity of variance by the Levene test. The significance level was 95% in all cases (*p* < 0.05). All the data were analysed by the computer application SPSS for Windows^®^ (version 15.0, SPSS Inc., Chicago, IL, USA).

## 5. Conclusions

For aquaculture application, we formulated a novel Gelucire^®^ 50/13/Precirol^®^ ATO5-based SLN intended as a colloidal vector for the administration of the antioxidant GSE to immunocompetent fish cells. The mild melt-emulsification technique provided particles that combined high AE% of GSE with a reduced crystallinity level regarding the organization of the solid state, as evidenced by X-ray diffraction and Raman spectra. From the biological evaluation of the SLNs, it was deduced, firstly, that both cell lines (SAF-1 and DLB-1) had different sensitivities to the exposure to the different GSE-SLNs at the concentrations and incubation times tested in the present study. Furthermore, our results demonstrated an important increase in antioxidant response (up-regulated expression of *Nrf2*, *cat*, *sod*, and *mt*) in the cells after being incubated with the GSE-SLN. The concentration of GSE, as well as the method of loading the GSE into the SLN, played crucial roles in the physiological effects on the SAF-1 and DLB-1 cell lines, as was demonstrated by the cytotoxicity assays and the gene expression of different apoptosis markers (*casp3* and *bax*). Finally, GSE-SLN affected the expression of different proteins related to the cytoskeleton and apoptosis, which evidenced that GSE-SLN can be considered as a compelling and valuable tool for use in different disease treatments. Moving on from good physicochemical characteristics, ex vivo results in terms of cell viability and the expression of different genes related to antioxidant defense, viability, and the cytoskeleton could be addressed in future studies to evaluate if SLNs containing GSE could be a candidate for in vivo trials in nutraceutical industries.

## Figures and Tables

**Table 1:** Physicochemical properties of GSE containing SLN. Mean ± standard deviations. are reported, *n* = 6. SLN without GSE (Unloaded SLN) were used as control for formulations. (**) *p* ≤ 0.001.

Formulation	Size (nm)	PDI	Zeta Potential (mV)	Association Efficiency (AE) (%)
Unloaded SLN	486(±5)	0.42–0.48	−32.7(±1.1)	−
GSE-SLN_(6mg)_	208(±21) **	0.44–0.49	−43.4(±1.8) **	49.7(±3.2)
GSE-SLN_(12mg)_	139(±15) **	0.44–0.48	−25.6(±2.8) **	64.9(±1.0)
GSE-SLN_(6mg)_-adsorbing GSE	283(±32) **	0.50–0.59	−43.0(±1.3) **	74.6(±0.2)

**Table 2:** Raman intensity ratios related to C-C stretching vibrational bands and to C-H stretching vibrational bands of analysed systems.

Sample	I_1115_/I_1050_	I_2890_/I_2850_
Precirol^®^ ATO5	1	1.4
Gelucire^®^ 50/13	1.02	1.67
Pure GSE	0.968	1.51
GSE-SLN_(6mg)_	0.867	1.83
GSE-SLN_(6mg)_-adsorbing GSE	0.562	1.84

**Table 3:** Total antioxidant activity of GSE-SLN.

Formulation	Total Antioxidant Activity (TAA) (eq Asc.) mM/mg Nanoparticles
GSE-SLN_(6mg)_	1.735 ± 0.327
GSE-SLN_(12mg)_	2.202 ± 0.321
GSE-SLN_(6mg)_-adsorbing GSE	1.411 ± 0.200

**Table 4:** Gilthead seabream primer sequences used for real-time PCR.

Gene	Accession Number	F/R Primer Sequence (5′–3′)
*nrf-2*	FP335773	F: GTTCAGTCGGTGCTTTGACA
		R: CTCTGATGTGCGTCTCTCCA
*sod*	AJ937872	F: CCATGGTAAGAATCATGGCGG
		R: CGTGGATCACCATGGTTCTG
*cat*	FG264808	F: TTCCCGTCCTTCATTCACTC
		R: CTCCAGAAGTCCCACACCAT
*hsp70*	EU805481	F: AATGTTCTGCGCATCATCAA
		R: GCCTCCACCAAGATCAAAGA
*bax*	AM963390	F: CAACAAGATGGCATCACACC
		R: TGAACCCGCTCGTATATGAAA
*casp3*	EU722334	F: CTGATCTGGATGGAGGCATT
		R: AGTAGTAGCCTGGGGCTGTG
*mt*	X97276	F: ACAAACTGCTCCTGCACCTC
		R: CAGCTAGTGTCGCACGTCTT
*vim*	FM155527	F: CGCTTACCTGTGAGGTGGAT
		R: GTGTCTTGGTAACCGCCTGT
*tub-a*	AY326430	F: AAGATGTGAACTCCGCCATC
		R: CTGGTAGTTGATGCCCACCT
*act-β*	X89920	F: GGCACCACACCTTCTACAATG
		R: GTGGTGGTGAAGCTGTAGCC
*18S*	AM490061	F: CTTCAACGCTCAGGTCATCAT
		R: AGTTGGCACCGTTTATGGTC

**Table 5:** European sea bass primer sequences used for real-time PCR.

Gene	Accession Number	F/R Primer Sequence (5′–3′)
*nrf2*	DLAgn_00051120	F: AACTAAGCCTCCCCTCACAC
		R: GTTGTGGTCCATCTCCTCCA
*sod*	FJ860004	F: TGTTGGAGACCTGGGAGATG
		R: ATTGGGCCTGTGAGAGTGAG
*cat*	FJ860003	F: GAGGTTTGCCTGATGGCTAC
		R: TGCAGTAGAAACGCTCACCA
*hsp70*	AY423555	F: CTGCTAAGAATGGCCTGGAG
		R: CTCGTTGCACTTGTCCAGAA
*bax*	FM011848	F: TGTCGACTCGTCATCAAAGC
		R: CACATGTTCCCGGAGGTAGT
*casp3*	DQ345773	F: AATTCACCAGGCTTCAATGC
		R: CTACGGCAGAGACGACATCA
*mt*	AF199014	F: GCACCACCTGCAAGAAGACT
		R: AGCTGGTGTCGCACGTCT
*vim*	FM018579	F: AGCGCCAGATTAGAGAGCTG
		R: GCCATCTCGTCCTTCATGTT
*tub-a*	AY326429	F: ACGAGGCCATCTACGACATC
		R: GGCCGTTATGGACGAGACTA
*act-β*	AJ537421	F: TCCCTGGAGAAGAGCTACGA
		R: AGGAAGGAAGGCTGGAAAAG
*18S*	AY831388	F: TTCCTTTGATCGCTCTTAACG
		R: TCTGATAAATGCACGCATCC

## Data Availability

The datasets generated during the current study will be available upon request.

## Sample Availability

Samples are not available from the authors.
